# Selection towards different adaptive optima drove the early diversification of locomotor phenotypes in the radiation of Neotropical geophagine cichlids

**DOI:** 10.1186/s12862-015-0348-7

**Published:** 2015-05-01

**Authors:** Viviana Astudillo-Clavijo, Jessica H Arbour, Hernán López-Fernández

**Affiliations:** Department of Ecology and Evolutionary Biology, University of Toronto, 25 Wilcocks St, Toronto, Ontario M5S 3B2 Canada; Department of Natural History, Royal Ontario Museum, 100 Queen’s Park, Toronto, Ontario M5S 2C6 Canada

**Keywords:** Cichlid, Locomotor morphology, Adaptive landscape, Adaptive peak, Early burst, Functional diversity, Comparative methods

## Abstract

**Background:**

Simpson envisaged a conceptual model of adaptive radiation in which lineages diversify into “adaptive zones” within a macroevolutionary adaptive landscape. However, only a handful of studies have empirically investigated this adaptive landscape and its consequences for our interpretation of the underlying mechanisms of phenotypic evolution. In fish radiations the evolution of locomotor phenotypes may represent an important dimension of ecomorphological diversification given the implications of locomotion for feeding and habitat use. Neotropical geophagine cichlids represent a newly identified adaptive radiation and provide a useful system for studying patterns of locomotor diversification and the implications of selective constraints on phenotypic divergence in general.

**Results:**

We use multivariate ordination, models of phenotypic evolution and posterior predictive approaches to investigate the macroevolutionary adaptive landscape and test for evidence of early divergence of locomotor phenotypes in Geophagini. The evolution of locomotor phenotypes was characterized by selection towards at least two distinct adaptive peaks and the early divergence of modern morphological disparity. One adaptive peak included the benthic and epibenthic invertivores and was characterized by fishes with deep, laterally compressed bodies that optimize precise, slow-swimming manoeuvres. The second adaptive peak resulted from a shift in adaptive optima in the species-rich ram-feeding/rheophilic *Crenicichla-Teleocichla* clade and was characterized by species with streamlined bodies that optimize fast starts and rapid manoeuvres. Evolutionary models and posterior predictive approaches favoured an early shift to a new adaptive peak over decreasing rates of evolution as the underlying process driving the early divergence of locomotor phenotypes.

**Conclusions:**

The influence of multiple adaptive peaks on the divergence of locomotor phenotypes in Geophagini is compatible with the expectations of an ecologically driven adaptive radiation. This study confirms that the diversification of locomotor phenotypes represents an important dimension of phenotypic evolution in the geophagine adaptive radiation. It also suggests that the commonly observed early burst of phenotypic evolution during adaptive radiations may be better explained by the concentration of shifts to new adaptive peaks deep in the phylogeny rather than overall decreasing rates of evolution.

**Electronic supplementary material:**

The online version of this article (doi:10.1186/s12862-015-0348-7) contains supplementary material, which is available to authorized users.

## Background

Simpson [1] conceptualized a model of adaptive radiation that includes a macroevolutionary adaptive landscape with a series of ecological “adaptive zones” that become occupied by diversifying lineages. Accordingly, an expectation that is frequently used to diagnose adaptive radiations is an early burst in phenotypic divergence, in which rates of divergence are predicted to decrease through time as lineages accumulate and niches associated with these “adaptive zones” become saturated [2,3]. Though support for an early burst model of phenotypic divergence is suggestive of adaptive niche filling, it does not allow us to assess the assumption of ecological selection or elucidate the “adaptive zones” of an adaptive radiation. Evolutionary models that incorporate selection along branches of the phylogeny [4-6] make it possible to investigate the macroevolutionary adaptive landscape more explicitly. The early burst model of divergence has been applied widely in comparative analyses of adaptive radiation (e.g. [7-10]), but a growing body of research is now also making use of evolutionary models based on a Simpsonian adaptive landscape (e.g. [11-14]).

Adaptive radiations generally involve phenotypic diversification along one or more ecological dimensions of specialization [2,4,15,16]. For example, the diversification of feeding strategies was accompanied by the diversification of bill morphology in Darwin’s finches [17], skull and mandible shape in phyllostomid bats [18], and head and oral jaw morphology in African [19] and Neotropical cichlids [20]. Similarly, habitat divergence was accompanied by the evolution of limb morphology in *Anolis* lizards [21].

In fish radiations the evolution of locomotor phenotypes (i.e. post-cranial attributes) may represent an important dimension of ecomorphological diversification. Fishes exhibit an extraordinary diversity of locomotor phenotypes that may have allowed them to diversify into an equally diverse array of ecological roles. Locomotion has major implications for ecological processes such as feeding and habitat use in fishes [22-24] because they live in a dense medium that requires swimming almost continuously. Different suites of locomotor traits optimize swimming performance for different ecological conditions [25-27] thus much of the observed diversity of locomotor phenotypes is likely related to ecological specialization. Relatively few studies have investigated the evolution of locomotor phenotypes (beyond overall body shape) in a phylogenetic comparative context [8,28] and as a result, our understanding of the patterns and processes of locomotor diversification and their importance in fish radiations remains limited.

**Figure 1:**
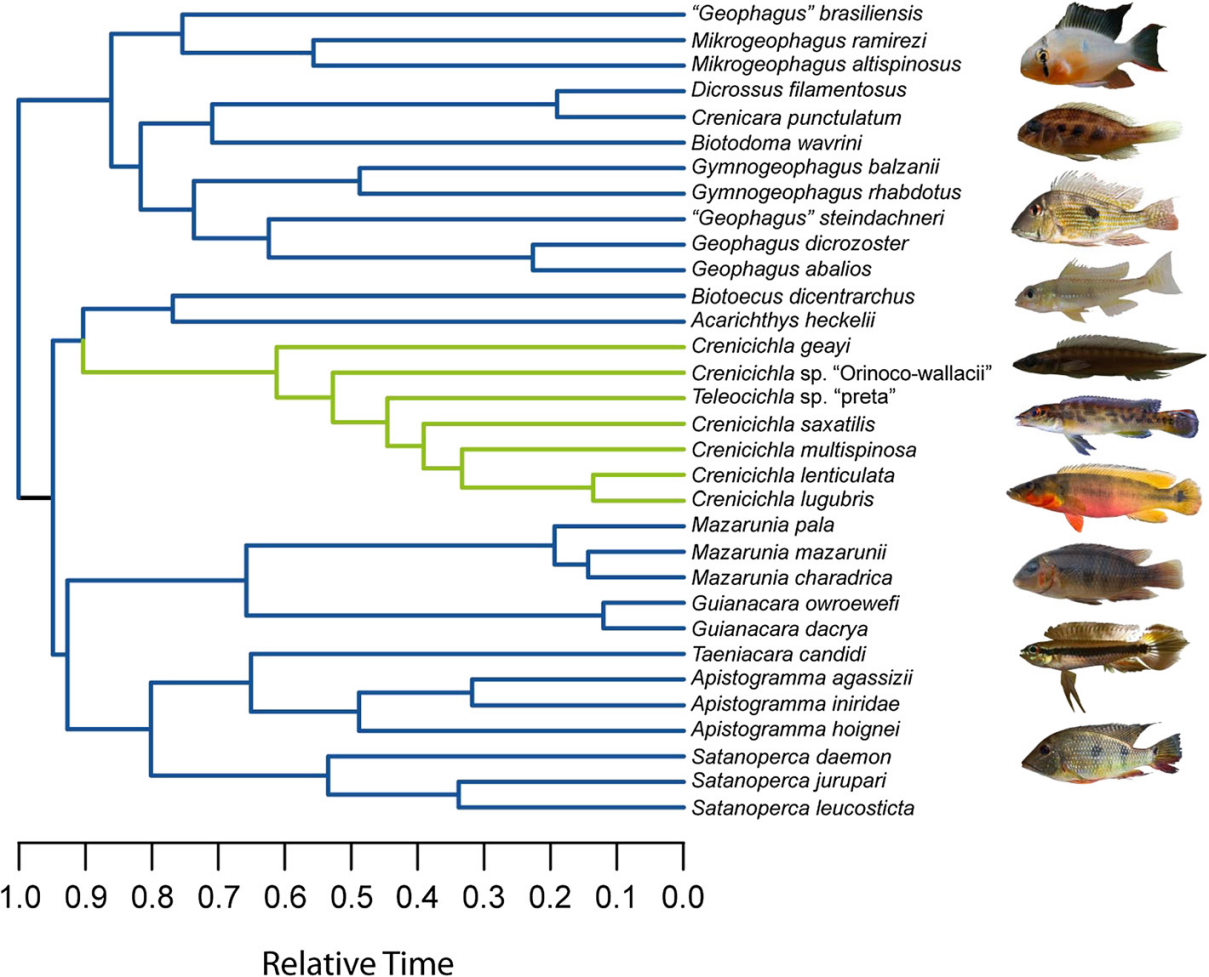
Geophagini Maximum Clade Credibility (MCC) tree. The MCC tree has been pruned to include species used in comparative analyses and scaled to a length of 1. *Crenicichla sveni* and *Teleocichla gephyrogramma* were used to approximate age and phylogenetic position of closely related *C. saxatilis and T.* sp. “preta” respectively which were not included in the original phylogeny. Photos are included to illustrate some of the phenotypic diversity in Geophagini. From the top photos are: *Mikrogeophagus altispinosus, Crenicara punctulatum, Geophagus* aff. *dicrozoster, Biotoecus dicentrarchus, Crenicichla* sp. “Orinoco-wallacii*, Teleocichla* sp. ‘preta’, *Crenicichla lugubris, Mazarunia charadrica, Taeniacra candidi,* and *Satanoperca daemon*. The coloured branches correspond to the geophagine adaptive peaks identified by the SURFACE model. Green branches: *Crenicichla-Teleocichla* adaptive peak. Blue branches: benthivorous/epibenthivorous adaptive peak. The MCC tree is modified from [23]. Photos were taken by H. López**-**Fernández, J.H. Arbour, K.M. Alofs, N.K. Lujan and C.G. Montaña.

Cichlinae (the Neotropical cichlids), and in particular its largest tribe Geophagini, offer the opportunity to study the diversification of locomotor phenotypes and to identify the underlying evolutionary mechanisms of phenotypic divergence during an adaptive radiation. Geophagini is the most diverse tribe of Neotropical cichlids with over 250 species in 17 genera that are phenotypically, ecologically and behaviourally diverse, with ecomorphological differentiation among genera exceeding intrageneric variation [9,29-31] (Figure 1). Several genera (*Acarichthys, Biotodoma, Geophagus, ‘Geophagus’ brasiliensis, ‘Geophagus’ steindachneri, Gymnogeophagus, Mikrogeophagus,* and *Satanoperca*), although distinct in overall morphology and feeding functional morphology [20] consist primarily of benthic substrate-sifting species that feed by extracting invertebrates from ingested substrate using a behaviour known as winnowing [31,32]. The ‘dwarf’ cichlids (*Apistogramma, Biotoecus, Crenicara,* some species of *Crenicichla*, *Dicrossus, Mikrogeophagus,* and *Taeniacara*) are small-bodied species that feed mostly on benthic and epibenthic invertebrates [21,33], often in structurally complex habitats, such as the leaf litter or woody debris [34]. The *Crenicichla-Teleocichla* clade is one of the most speciose and ecomorphologically unique geophagine lineages with at least 90 described species of primarily elongate-bodied piscivore and insectivore *Crenicichla* species and various elongate-bodied rheophilic *Teleocichla* species [30,31,34-36].

Recent work showed that Geophagini represents an ancient continent-wide adaptive radiation [9]. Extensive work on feeding functional morphology [20] and trophic divergence [29,31-33] identified trophic specialization as a major source of adaptive diversification in the geophagine radiation with predators, benthic sifters, and invertebrate pickers exhibiting trait complexes associated with strong biting, suction, and rapid jaw-closing capabilities respectively [20]. A similar detailed investigation into the evolution of locomotor morphology is currently lacking. Developing an understanding of diversification along multiple functional morphological dimensions in Geophagini will provide us with a clearer picture of the ecological and evolutionary processes involved in the adaptive radiation of one of the most diverse groups of Neotropical fishes.

In this study we used a multivariate analysis of functional traits from a sample of 32 species and a time**-**calibrated phylogeny to look at the evolution of locomotor morphology in Geophagini. We investigate whether there is evidence of adaptive divergence and whether the inclusion of a model that incorporates selective constraints akin to the macroevolutionary adaptive landscape provides a clearer interpretation of the underlying mechanisms of locomotor diversification. We use multivariate ordination and models of phenotypic divergence to (1) assess patterns of locomotor morphological diversity amongst lineages, (2) investigate the adaptive landscape, and (3) test for an early burst of phenotypic diversification. Our findings confirm that locomotion was an important dimension of phenotypic divergence during the geophagine adaptive radiation. They further emphasize that a model that incorporates the macroevolutionary adaptive landscape provides a better-fitting and perhaps biologically more informative account of the historical underlying mechanisms of phenotypic evolution in adaptive radiations.

## Results

### Patterns of morphological diversity

**Figure 2:**
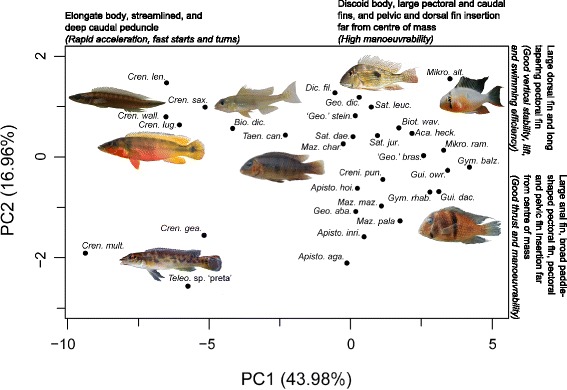
Phylogenetic Principal Components Analysis (PCA) for locomotor morphology based on the Maximum Clade Credibility (MCC) tree. Refer to Figure 1 for complete species names. The green and blue convex hulls indicate the areas of morphospace occupied by lineages belonging to the two geophagine adaptive peaks as identified by SURFACE. Green: *Crenicichla-Teleocichla* adaptive peak. Blue: benthivorous/epibenthivorous adaptive peak. Colours correspond to those on Figure 1. Text on the top and right margin of the plot indicate the trait complexes and functional implications of these trait complexes based on published literature (refer to text and Additional file 2 for details) at the extremes of PC1 and PC2. Numbers in brackets indicate the percent variance explained by each of the critical PC axes. Photos are included in the plot to show the variation in functional morphology along the axes and correspond to species present near the extreme of each axis. Starting with the photos at the top left corner and going clockwise, the species represented are: *Crenicichla* sp. “Orinoco-wallacii”*, Geophagus* aff. *dicrozoster, Mikrogeophagus altispinosus, Guianacara dacrya, Teleocichla* sp. “preta”, and *Crenicichla lugubriscp* The photo in the centre of the plot is *Mazarunia charadrica*. Photos were taken by H. López**-**Fernández, J.H. Arbour, K.M. Alofs, N.K. Lujan.

We investigated the diversity of locomotor attributes in Geophagini by performing a phylogenetically-corrected Principal Components Analysis (PCA) using a sample of 1000 posterior distribution chronograms for 32 species that span the crowns of the major ecomorphological subclades within Geophagini. The PCA recovered two critical Principal Component (PC) axes, which together accounted for an average of 61.04 +/− 0.78% of the variation in swimming morphology across 1000 posterior distribution trees (Table 1, Figure 2). PC1 explained on average 43.80 +/− 0.33% of the variation and was most strongly influenced by variables that describe body shape, pectoral and caudal fin area, and pelvic and dorsal fin position. Species near the negative extreme had shallow streamlined bodies with a high fineness ratio and a caudal peduncle almost as deep as the maximum body depth (elongate body). *Crenicichla* and *Teleocichla* had fineness ratios from 4.5**-**6.7, which is within the optimal streamlining range (e.g. [27]; see Additional file 1). The *Crenicichla-Teleocichla* clade occupied a large region of morphospace that excluded all non-*Crenicichla-Teleocichla* species. In contrast, species near the positive extreme included most of the benthic and epibenthic species that were characterized by a deep laterally-compressed body (discoid body), large frontal area, large pectoral and caudal fins, and large pelvic and dorsal moment arms. PC2 explained on average 17.24 +/− 0.66% of the variation and was most strongly influenced by variables that describe pectoral fin shape, median fin areas, and the position of the paired fins relative to the centre of mass. High anal fin area, a low aspect ratio pectoral fin, and a long pectoral fin moment arm characterized species near the negative extreme of PC2, while those at the positive extreme had a high dorsal fin area and a high pectoral fin aspect ratio.

**Table 1:** **Mean +/− standard deviation of eigenvectors, eigenvalues, and variance across the 1000 chronogram set**

	**PC1**	**PC2**
Body depth	**0.38 +/− 0.00**	−0.08 +/− 0.02
Body width	**−0.32 +/− 0.01**	−0.10 +/− 0.05
Pectoral fin moment arm	−0.06 +/− 0.02	**−0.47 +/− 0.05**
Pelvic fin moment arm	**0.25 +/− 0.01**	**−0.33 +/− 0.04**
Anal fin moment arm	0.09 +/− 0.01	0.21 +/− 0.06
Dorsal fin moment arm	**0.29 +/− 0.01**	0.13 +/− 0.04
Pectoral fin area	**0.31 +/− 0.00**	−0.15 +/− 0.03
Anal fin area	0.07 +/− 0.02	**−0.48 +/− 0.04**
Dorsal fin area	0.11 +/− 0.03	**0.42 +/− 0.06**
Caudal fin area	**0.30 +/− 0.01**	−0.00 +/− 0.02
Pectoral fin aspect ratio	0.09 +/− 0.02	**0.36 +/− 0.04**
Caudal fin aspect ratio	0.08 +/− 0.01	0.04 +/− 0.04
Peduncle:Body depth ratio	**−0.33 +/− 0.00**	−0.13 +/− 0.02
Frontal area	**0.36 +/− 0.00**	−0.07 +/− 0.02
Fineness ratio	**−0.38 +/− 0.00**	0.08 +/− 0.02
Eigenvalue	6.57 +/− 0.10	2.59 +/− 0.10
% variance	43.80 +/− 0.66	17.24 +/− 0.66

Bolded values correspond to eigenvectors whose confidence intervals do not overlap with those of the eigenvectors for the 1000 permuted datasets.

### Macroevolutionary adaptive landscape

SURFACE models [37] were fit for PC1 and PC2 to investigate the macroevolutionary adaptive landscape of Geophagini. The best supported SURFACE model for PC1 included a single regime shift at the base of the *Crenicichla-Teleocichla* clade, which produced a model with 2 non-convergent adaptive peaks in the *Crenicichla-Teleocichla* clade and the remaining benthic and epibenthic lineages (mean +/− SD of model parameters: α = 0.974 +/− 0.07, σ^2^ = 7.420 +/− 0.12, θ_Cren.-Teleo_ = −11.027 +/− 1.65, θ_benth./epibenth._ = 1.126 +/− 0.20; Figure 2). The number of peaks and incidences of convergence resulting from Brownian Motion (BM) simulation models were not significantly different from those of the SURFACE model (Table 2), suggesting that a pattern consistent with 2 adaptive peaks could have arisen in Geophagini under a random walk process. The best SURFACE model for PC2 was one with a single adaptive peak for all geophagine cichlids (mean +/− SD of model parameters: α = 14.032 +/− 22.87 (median = 6.77), σ^2^ = 32.838 +/− 53.507 (median = 15.84), θ_all_ = −0.088 +/− 0.01).

**Table 2:** **SURFACE parameters for PC1 and Brownian motion (BM) simulations**

**SURFACE parameters**	**SURFACE**	**BM**	**P**
**K**	2	3.18 +/− 1.14	0.95
**k’**	2	2.70 +/− 0.88	0.95
**C**	0	0.92 +/− 0.17	1.00
**k’**_**conv**_	0	0.43 +/− 0.55	1.00
Δ**k**	0	0.48 +/− 0.65	1.00
**c/k**	0	0.23 +/− 0.99	1.00

BM values represent the mean +/− standard deviation of SURFACE parameter estimates across the 500 BM simulations of the morphological data. k = number of peaks before convergence; k’ = number of non-convergent peaks; c = number of shifts to convergent peaks; k’_conv_ = number of convergent peaks; Δk = reduction in the number of peaks with convergence; c/k = the proportion of convergent peaks relative to the total number of peaks. P = the proportion of BM simulations that produced SURFACE parameters as high or higher than the preferred SURFACE model for PC1.

### Early accumulation of morphological disparity

We tested for evidence of an “early burst” in locomotor divergence using maximum likelihood models and disparity through time (DTT) analyses [38]. BM, OU, Early Burst (EB), and SURFACE models were fit and compared for PC1 and PC2 across the 1000 posterior distribution trees and then used to simulate the expected pattern of divergence under each model. Based on the maximum likelihood approach, the evolution of PC1 was best described by a 2-peak Ornstein-Uhlenbeck (OU) model (i.e. the SURFACE model), while the evolution of PC2 corresponded to a single-peak OU model. Both OU and SURFACE models produced identical results for PC2 (i.e. a single adaptive peak for the entire clade), and thus here we compare only BM, OU, and EB models for this axis (Table 3).

**Table 3:** **Brownian Motion (BM), Ornstein-Uhlenbeck (OU), Early Burst (EB), and SURFACE model summaries**

		**BM**	**OU**	**EB**	**SURFACE**
**PC1**	AIC	141.18+/− 1.25	143.62 +/−1.25	138.80 +/− 1.93	129.790 +/− 0.94
	ΔAIC	11.385 +/− 4.51	13.83 +/− 4.51	9.01 +/− 4.49	0.00 +/− 0.0
	wAIC	0.012 +/− 0.01	0.003 +/− 0.00	0.04 +/− 0.047	0.95 +/− 0.06
**PC2**	AIC	111.953 +/− 1.82	105.42 +/− 0.23	114.40 +/− 1.82	**_**
	ΔAIC	9.502 +/− 1.72	0.00 +/− 0.00	11.95 +/− 1.72	**_**
	wAIC	0.041 +/− 0.03	0.95 +/− 0.045	0.01 +/− 0.01	**_**

AIC: Akaike Information Criterion, ΔAIC: the difference in AIC between each model and the best model (AIC of the best model = 0), wAIC: the weight of support for each model across 1000 posterior distribution chronograms. Values represent the mean ± standard deviation across 1000 posterior distribution trees.

**Figure 3:**
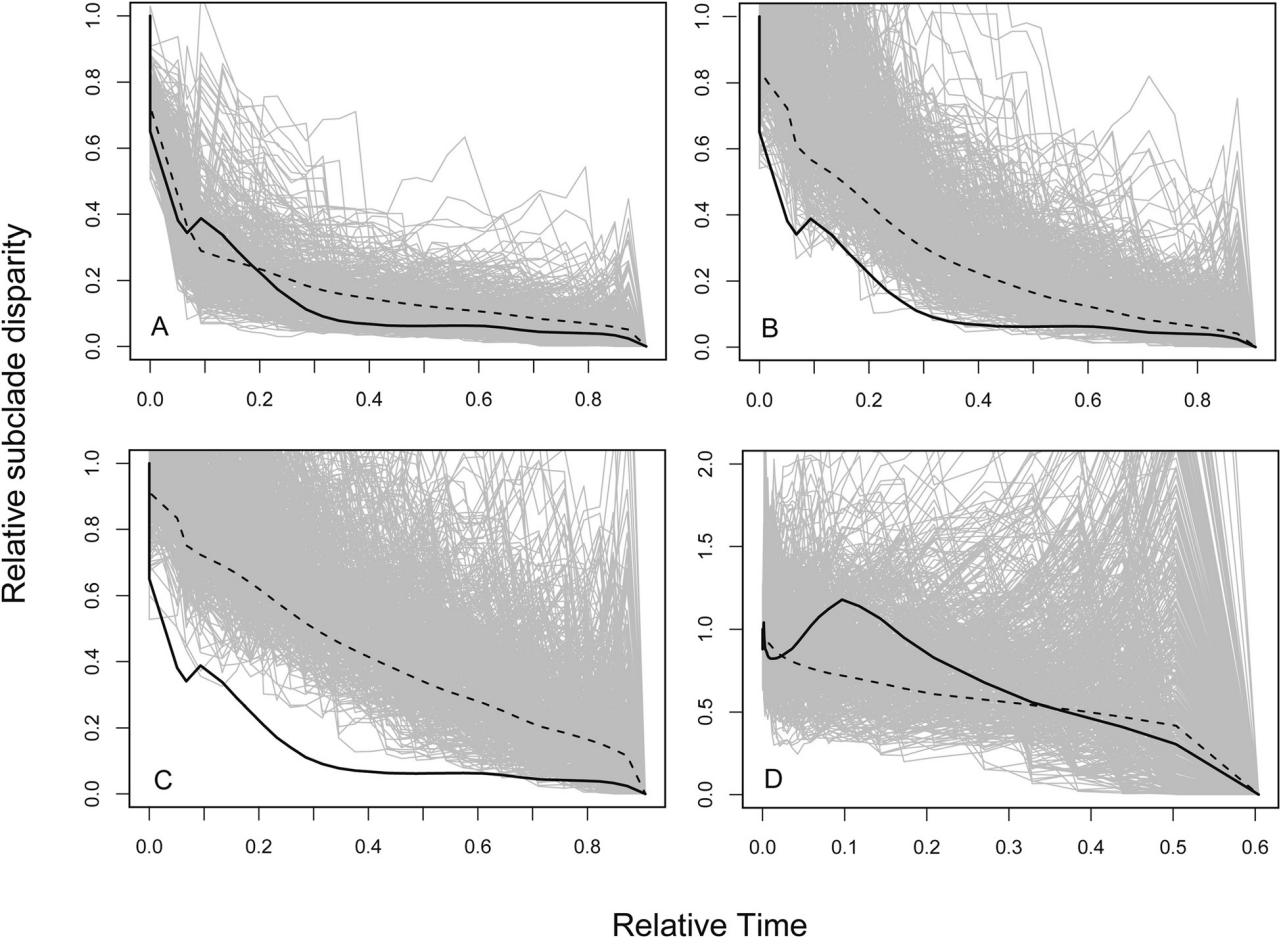
Disparity through time (DTT) plots for PC1 and PC2 axes. Grey lines show a random subset of 10 000 Brownian motion (BM) simulations that fall within the 95% confidence interval of the 1000 simulations performed for each of the 1000 posterior distribution trees. The dotted line is the mean change in disparity across all simulations and the solid black line shows the mean of the actual change in disparity across the 1000 trees. The observed DTT curve for PC1 was compared to simulations under the **A)** SURFACE model, **B)** early burst model, and **C)** BM model. **D)** The observed DTT curve for PC2 was compared to simulations under a single-peak Ornstein Uhlenbeck model.

DTT analyses generally corroborated the results of the maximum likelihood approach. Simulations under the best model for PC1 and PC2, according to the maximum likelihood approach, produced similar patterns of divergence as the observed data. The mean morphological disparity index (MDI; area between simulated and observed DTT curves) and posterior predictive p (henceforth p; frequency of simulated curves as extreme as the observed DTT curve) for DTT based on SURFACE simulations across the 1000 trees were −0.016 +/− 0.0017 and 0.24 +/− 0.10 respectively (Figure 3A). Though maximum likelihood also rejected an EB model for PC1, DTT showed that the observed pattern of divergence differed only slightly from the expected pattern under an EB model (mean MDI = −0.088 +/− 0.019, mean p-value = 0.088 +/− 0.055; Figure 3B). Both maximum likelihood and DTT rejected a BM model of phenotypic evolution for PC1 (Figure 3C). DTT analyses for PC1 based on BM simulations resulted in a mean MDI value of −0.20 +/− 0.018 and mean p-value of 0.0034 +/− 0.00. Thus the observed MDI value for PC1 was best approximated by a two-peak OU model (highest p-value; [39]) with some support for an EB model. Both SURFACE and EB models suggest that the accumulation of morphological disparity decreased precipitously near the base of the tree, after which later divergence events contributed little to the overall morphological disparity of the clade. DTT for PC2 analyses based on single-peak OU simulations resulted in a mean MDI value of 0.12 +/− 0.056 and mean p-value of 0.21 +/− 0.11 (Figure 3D).

## Discussion

The phylogenetic PCA recovered two critical PC axes consistent with combinations of locomotor traits that characterize adaptation to divergent swimming modes [see Additional file 1]. Most of the locomotor phenotypic diversity in Geophagini is related to traits associated with PC1. PC1 represents a gradient from elongate-bodied fishes with a deep caudal peduncle to discoid-bodied fishes with large pectoral and caudal fins and pelvic and dorsal fins positioned relatively far from the center of mass. Trait complexes at the extremes of PC1 appear to be related to a trade-off between adaptations for fast starts and high-speed manoeuvres (negative end of axis) and for precise slow-swimming manoeuvres (positive end of axis). A streamlined body and deep caudal peduncle are optimal for fast starts and rapid turns and manoeuvres because this phenotype concentrates thrust near the trailing edge of the fins and provides the largest contribution to overall thrust and momentum [25]. In contrast, a discoid body with large median fins positioned far from the center of mass is better adapted for performing precise slow-swimming manoeuvres. A discoid body allows for tight turns by reducing the vertical turning radius [25,40]. Large fins and fins positioned far from the centre of mass increase the volume of water that is moved and the torque or force applied to move that water respectively at specific regions of the body, which allows for localized and precisely directed acceleration, turning, breaking, and balancing manoeuvres [22,25,27,40,41].

The distribution of species along PC1 reflects an early adaptive peak shift in the *Crenicichla-Teleocichla* lineage that resulted in selection towards a ram/rheophilic adaptive peak in the *Crenicichla-Teleocichla* lineage and a benthic/epibenthic adaptive peak in the remaining geophagine lineages (the preferred SURFACE model). The negative extreme of PC1 corresponds to the ram/rheophilic adaptive peak and is occupied exclusively by members of the *Crenicichla-Teleocichla* clade. These species are active, ram-feeding predators (*Crenicichla*) and/or rheophilic specialists (*Teleocichla*) [31,35] that benefit from having a streamlined body with a deep caudal region for ambushing prey or manoeuvring in strong river currents. The positive extreme of PC1 corresponds to the benthic/epibenthic adaptive peak and includes all of the non-*Crenicichla-Teleocichla* lineages. Species at the positive extreme of PC1 are benthic substrate-sifters and benthic/epibenthic invertebrate pickers that feed on slow or non-evasive prey [31,32] and thus may rely greatly on the optimization of precise, slow swimming manoeuvres. Precise manoeuvring in the lower levels of the water column is an integral part of the widespread behaviours involved in picking up substrate into the mouth cavity and processing it through winnowing (e.g. [42]). Neotropical cichlid lineages that feed by substrate-sifting have been found to be highly convergent functionally, despite a relatively large amount of overall morphological diversity [32]. Results from the present study suggest that morphological variation in body shape and other locomotor attributes within substrate sifters (positive end of PC1) may reflect yet undescribed variation in locomotor function that may be associated with fine-tuning of habitat use. Further studies should address the correlation between locomotor attributes found herein and variables that describe habitat attributes.

BM simulations suggest that a single peak shift in PC1 could have also arisen under a random-walk process (Table 2), but maximum likelihood model comparisons strongly rejects BM in favour of a 2-peak OU model (Table 3). The probability of arriving at similar regions of trait space under a random-walk process increases with decreasing number of taxa [43]. It is possible that the inclusion of a larger number of taxa in future work will recover more adaptive peaks that outnumber those expected under a BM process and provide more definitive support for the validity of the *Crenicichla-Teleocichla* adaptive peak (e.g. [14]). Work on the ecology [31,33], functional morphology [20,31], and evolution [14,38] of Neotropical cichlids provide additional support for a *Crenicichla-Teleocichla* adaptive peak. The *Crenicichla-Teleocichla* clade also occupied a unique region of trophic morphospace that corresponded to an adaptive peak characterized by fast protrusible jaws capable of strong gripping bites that optimize ram feeding [20,14]. It appears that the *Crenicichla-Teleocichla* lineage represents a singular case of morphological innovation in both locomotion and feeding that, early in geophagine diversification, moved the clade into a new selective regime that favoured exploration of new ecological niches not available to the remaining geophagine lineages. The large taxonomic and functional diversity of the *Crenicichla-Teleocichla* clade suggests that further evolutionary diversification occurred within the group, a suggestion further supported by the finding of a so-called “species flock” of *Crenicichla* in the Paraná River basin of South America [44].

PC2 represents a gradient of slight variation in the size shape and position of paired and median fins. Paired and median fins have functional implications for manoeuvrability, and thus variation along PC2 appears to be related the diversity of locomotor phenotypes that optimize manoeuvrability under different conditions. For example, substrate sifters near the positive end of PC2 spend considerable time sifting for invertebrates in sandy substrates adjacent to structurally complex rocky or vegetated habitats into which they retreat periodically ([45], HLF pers obs). During sifting their large dorsal fin may help them maintain a vertical position in the water column by producing balancing torques that reduce the tendency to roll or yaw [46]. Additionally, their high aspect ratio paired fins (tapering fins) are optimal for generating lift and reducing drag [26,47,48], which may be an adaptation for maximizing swimming efficiency and hovering close to the substrate while sifting. Taxa near the negative end of PC2, such as *Apistogramma*, *Teleocichla*, and a few *Crenicichla* species, are characterized by pectoral fins with a slightly lower aspect ratio (broad, paddle-shaped fins) and longer moment arms. Low aspect-ratio fins generate more thrust during the power stroke than tapering fins, especially at low speeds [26,48], and have been found in species that remain in close proximity to structurally complex habitats [49]. Similarly, *Apistogramma* and *Teleocichla* are associated with highly structured habitats such as the leaf litter and rocky crevices respectively [34,44,50]. Though species exhibit some variation along PC2, the reduced variation relative to PC1 and the preference of a single-peak OU model suggest that the evolution of paired and median fin attributes, at least as described herein, is restricted to a single adaptive optimum in all of Geophagini.

All of the traits loading strongly on PC2 are related to the paired and median fins, which are functionally relevant for slow-swimming manoeuvres because the distribution and mobility of the paired and median fins allow fish to generate small, localized, and specifically directed bouts of propulsive thrust [22,25,27,40,41]. Paired/median fin propulsion is important for navigating tight turns and crevices in structurally complex habitats [22]. Species belonging to both the ram/rheophilic and benthic/epibenthic adaptive peaks are associated with structured habitats to some extent, which may have constrained the evolution of some paired and median fin attributes to a small range of phenotypes that improve manoeuvrability in structurally complex habitats. Therefore the apparent functional trade-offs of PC1 and convergence of PC2 may be due to selection towards either a ram/rheophilic or benthic/epibenthic adaptive peak within the selective confines of structurally complex habitats.

The EB and our multi-peak OU (SURFACE) model predicted decreasing phenotypic divergence in PC1, but both maximum likelihood (Table 3) and posterior predictive approaches (Figure 3A,B) suggested that the observed accumulation of phenotypic disparity approaches the early divergence pattern produced by the multi-peak OU model more closely than that of the EB model. EB assumes that decreased phenotypic divergence towards the tips is due to decreasing rates of divergence, presumably as a result of niche saturation by early adapting lineages [7]. Our multi-peak OU model assumes that decreased divergence near the tips is due to evolution towards different adaptive optima that were adopted by early diversifying lineages. Though both models suggest that decreasing divergence is related to early adaptive diversification, the observed divergence patterns were likely better fit by the multi-peak OU model because by incorporating multiple selective regimes it provides a more explicit and biologically realistic account of adaptive divergence along the branches of the phylogeny. Most DTT analyses used to test for evidence of adaptive radiation compare the observed pattern only to the expected pattern under a constant rate BM process, or sometimes under an EB process [39], and interpret deviations from BM as evidence for decreasing rates of evolution. Here we show that a multi-peak OU model akin to a Simpsonian macroevolutionary adaptive landscape with early adaptive peak shifts may better explain the commonly observed “early burst” of adaptive radiations.

DTT further suggest that the utility of adaptive landscape models in describing phenotypic diversification extends beyond the identification of the best-fit model. Comparative datasets contain a wealth of information about the evolutionary process that is only partially tapped with traditional model fitting approaches. Despite the overlap in SURFACE parameter estimates for BM simulations and our 2-peak OU model (Table 2), the application of the latter to posterior-predictive analyses revealed an early divergence pattern underlain by adaptive optima shifts (Figure 3) that is undetectable with model fitting alone (Table 3). Previous analyses have also shown that traditional model fitting approaches may be limited in their power to characterize adaptive processes in trait evolution [39]. Evaluation of phenotypic change over time, as done using DTT analyses, provides a more nuanced and detailed representation of the evolutionary patterns than that obtained by model-fitting approaches. Altogether, our results suggest that complementing model fitting methods with adaptive landscape perspectives provides a richer, more informative understanding of clade-wide divergence patterns and their possible underlying evolutionary processes.

The early diversification of locomotor morphology in Geophagini is consistent with the role of ecological opportunity and niche filling in phenotypic evolution within the clade. The unique *Crenicichla-Teleocichla* adaptive peak may be the result of a key innovation that presented the lineage with new ecological opportunity not available to or being exploited extensively by other taxa, which may have in turn promoted its extensive diversification into over 90 species. Subsequent competition between increasingly species-rich geophagine lineages likely contributed to the lack of more recent shifts between adaptive peaks in the evolution of Geophagini. Divergence of benthic and pelagic predatory/zooplanktivorous ecomorphs has been reported in several marine [51,52], lacustrine [53,54], and riverine lineages [55]. Furthermore, an early benthic-pelagic shift resulted in a burst in diversification during the adaptive radiation of an eastern North American cyprinid clade [55]. It appears that evolution along a benthic-pelagic axis has been an important source of diversity across distantly related lineages and different aquatic ecosystems.

Though our sample of taxa spans the crowns of the major ecomorphological subclades, the relatively small sample size (32 species) may have prevented SURFACE from identifying more complex models with additional adaptive peaks due to the lack of power to reject simpler models [40]. Nonetheless, geophagine species exhibit high levels of intrageneric similarity in ecology [31,33,34] and morphology [31]. Additionally, ~50 million year old *Gymnogeophagus* fossils have been found to occupy the same region of morphospace as modern representatives of the genus [Arbour and López-Fernández unpublished]. Thus we hypothesize that if additional adaptive peaks do exist in Geophagini, shifts towards these peaks will also be concentrated deep in the phylogeny and will thus continue to support a multi-peak OU model as the most likely explanation of the observed early burst pattern.

Considerable variation exists among estimates of cichlid ages, with estimates ranging from Eocene [56,57] to late Cretaceous [58] to late Jurassic-early Cretaceous [30] origin. All of these estimates are problematic and must be considered tentative because some suggest ages that are likely too old (e.g. [9]) and others find the family to be younger (e.g. [57]) or barely older than its own crown-lineage fossils (e.g. [57]). The set of 1000-scaled trees used in this study were based on absolute age estimates that date the origins of Cichlidae to 150 Mya (95% highest posterior density 128.2–174.78, 9). Even in relative time trees like those used in this study, differences in age estimates could affect internode distances, which in turn could affect the early burst pattern observed here. Fossil evidence constrains the origin of the geophagine genus *Gymnogeophagus* to a conservative estimate of at least 39.9 million years old, although it is likely older [9,59,60]. Younger ages for the family combined with older ages for crown lineages would compress basal branches by virtue of reducing the time from initial divergence to attaining recognizable modern diversity. Shorter basal branches derived from younger family-ages than the ones reflected in the branch lengths used in our study would strengthen the early burst signal detected here, and thus this work provides, at worst, a conservative estimate of early phenotypic divergence in Geophagini.

## Conclusions

Cichlinae is amongst the most speciose and ecologically diverse groups of fishes in the Neotropics and recently studied patterns of phyletic and phenotypic diversification suggest this diversity resulted from at least one episode of continent**-**wide adaptive radiation. Extensive work on trophic adaptive diversification has shown that the evolution of trophic phenotypes was an important dimension of ecomorphological diversification during the geophagine radiation; our study offers a complementary look at the diversification of locomotor morphology. Variation of locomotor trait complexes in geophagine cichlids suggest that post-cranial phenotypic diversity may be related to adaptations for feeding and habitat use. Much of the modern diversity in locomotor attributes arose during an early burst of morphological diversification. Evolutionary model fitting and disparity analyses favoured evolution towards different adaptive peaks as the underlying mechanism driving the early burst of locomotor diversification. These results suggests that perhaps the commonly observed early burst of adaptive radiations is better explained by a multi-peak OU model akin to a Simpsonian macroevolutionary adaptive landscape with early adaptive peak shifts than the traditional EB model, which emphasizes the potential role of selective constraint in shaping adaptive radiations. A hypothesis of the phylogenetic position of adaptive peak shifts also provides a framework for future assessments of the ecological context of phenotypic divergence in adaptive radiations.

## Methods

### Phylogenetic relationships and divergence times

Analyses were carried out on a sample of 1000 posterior distribution Cichlinae chronograms from López**-**Fernández et al. [9,36] to account for topological and branch length uncertainty. Cichlinae chronograms were obtained using relaxed molecular clock methods and Bayesian inference based on the alignment of 3868 base pairs from two nuclear and three mitochondrial genes for 166 cichlid species in BEAST 1.6.2. The Maximum Clade Credibility (MCC) tree and 1000-chronogram set were pruned to include only the species for which morphological data were collected, and scaled to a total length of 1 to account for total length differences and make results comparable across trees ([61]; see 9,36 for details of phylogenetic reconstruction and chronogram generation).

### Taxon sampling and morphological data

We measured standard length (SL) and 15 functional locomotor attributes related to swimming performance on preserved museum specimens housed at the Royal Ontario Museum (ROM), Canada representing each of the geophagine genera [61]. A total of 111 specimens from 32 species were measured with 1–6 individuals per species (average 3.5) and 1–6 species per genus (average 1.9). Only adult individuals with similar intraspecific SL values were used to minimize potential variance due to ontogenetic allometry [Additional file 2]. The inclusion of all genera in the tribe ensures that all crown lineages are considered in our analyses. Moreover, inclusion of a few species per genus should provide adequate sampling of the crowns of the major ecomorphological subclades. Species belonging to the same genus exhibit similar diets [31,33], habitat preferences [34], and morphologies [20,31,33,34], and intergeneric morphological variation vastly exceeds intrageneric variation [31]. We strived to sample morphological attributes of the same species included in the phylogeny, however, as specimens were not available for *Crenicichla sveni* and *Teleocichla gephyrogramma*, they were replaced with data from the related *C. saxatilis* and *T.* sp “preta”, respectively, for comparative analyses.

Locomotor attributes included body depth, body width:depth ratio, moment arms of the paired (pectoral and pelvic) and median (dorsal and anal) fins, surface area of the pectoral, caudal, and median fins, aspect ratio of the pectoral and caudal fins, peduncle:body depth ratio, frontal area, and fineness ratio. Linear measurements were taken directly on the fish using digital calipers and areas were measured on photographs of the corresponding measured individuals using the program ImageJ [62] [see Additional files 2 and 3 and references therein]. Following Gerry et al. [63], each fish’s left side was photographed with fins spread and pinned down keeping the shot angle perpendicular to the lateral plane of the fish. To ensure the specimen lay in a horizontal plane, photographs were taken with the fish laid on a foam board with a groove carved out to accommodate the curvature of the body. The foam board was placed over a grid that was used to calibrate measurements taken on photographs. Each specimen was photographed in front of a mirror positioned at a 45° angle with respect to the horizontal plane of the fish, providing a simultaneous lateral and frontal view of the fish in each photo. To find the centre of mass, each fish was photographed 3 more times while hanging from a string attached to a different fin (dorsal, anal, and caudal) each time. These photographs were superimposed on each other and rotated to match the margins of the fish shape in Adobe Photoshop CS5^©^. The point of intersection of the string in the 3 overlain photographs was determined to be the centre of mass of each individual [63].

### Patterns of morphological diversity

SL and locomotor attributes were log**-**transformed to standardize trait scales and make interspecific variance comparable for large and small specimens. Species means were obtained for each log**-**transformed variable. Locomotor attributes were corrected for size by regressing each mean log-transformed locomotor attribute against mean log-transformed SL and obtaining the residuals. Phylogenetically-corrected Principal Components Analyses (PCA) were performed on the residuals for each of the 1000 posterior distribution trees and for the MCC tree. Size-correction and phylogenetic PCAs were carried out using the functions “phyl.resid” and “phyl.pca” in the R package “phytools” [64]. Critical Principal Component (PC) axes and the morphological attributes that influence critical PC axes most strongly were identified by permuting the original morphological dataset 1000 times and performing a phylogenetic PCA on each of these permuted datasets. We compared the mean and 95% confidence interval (CI) of eigenvalues and eigenvectors across the 1000 trees to the mean and 95% CI of the eigenvalues and eigenvectors for the permuted datasets. Similar to Horn’s parallel analysis [65], we considered critical PC axes to be those with eigenvalue distributions greater than the distribution of permuted eigenvalues. Morphological attributes with eigenvector distributions that did not overlap with those of the permuted datasets were considered important descriptors of interspecific variability in the corresponding axes.

### Macroevolutionary adaptive landscape

To investigate the macroevolutionary adaptive landscape of locomotor morphology in Geophagini we tested for shifts in selective regimes that yield new adaptive peaks across lineages using PC1 and PC2 scores. We used Ingram and Mahler’s [37] SURFACE algorithm, which searches for adaptive peaks and convergence on those peaks without a-priori identification of lineages in which regime shifts may have occurred. SURFACE searches the adaptive landscape by fitting a series of increasingly complex Ornstein-Uhlenbeck (OU) models. SURFACE carries out a “forward” search phase, during which adaptive peaks are added to various locations in a phylogeny until there is no additional improvement in AIC scores, followed by a “backward” phase, during which similar adaptive peaks are collapsed together.

SURFACE models were fit for PC1 and PC2 on the sample of 1000 posterior distribution trees. PC1 and PC2 were analyzed independently because traits associated with these axes were characterized by different modes of evolution (see Results and Table 3) and a combined PC1-PC2 analysis does not change the geophagine adaptive landscape (not shown). We also assessed the effect of a larger morphospace (up to 4 PC axes) given the potential consequences of underestimating the number of critical PC axes on macroevolutionary patterns due to the loss of relevant information with discarded axes. Additional PC axes had no effect on the macroevolutionary adaptive landscape (results not shown) and are not discussed further.

We simulated the evolution of locomotor morphology under a Brownian motion (BM) model 500 times for the PC axis that was best fit by a SURFACE model to see if the same number of adaptive peaks could have also resulted from a random-walk process [37]. SURFACE models were fit for each of these simulated datasets and the number of regime shifts produced by the SURFACE models for the observed and simulated datasets were compared. Given the computational intensity of the SURFACE algorithm, the BM simulations could not be performed on the 1000 trees and thus BM simulations and the comparison of simulated and observed regime shifts were performed only for the MCC tree. SURFACE model fitting and BM simulations were implemented using the functions “runSurface” and “surfaceSimulate” respectively in the R package “surface” [37].

### Early accumulation of morphological disparity

We used maximum likelihood and disparity through time (DTT) analyses [38] to test whether Geophagini experienced an early burst in the diversification of locomotor attributes. The DTT approach differs from the maximum likelihood approach in that rather than choosing the single best model that maximizes the probability of the observed data it allows us to compare the observed pattern of phenotypic divergence to expected patterns of divergence under different evolutionary models using simulations [39].

The fit of the best SURFACE model was compared with the fit of BM, single-peak OU, and early burst (EB) models of PC1 and PC2 evolution across the 1000 posterior distribution trees using ΔAIC, and the Aikaike weight of evidence [66]. A BM model assumes that morphological evolution has occurred under a random walk process in which morphological disparity accumulates approximately linearly through time. A single-peak OU model assumes that morphological evolution has been constrained toward a single adaptive peak [4,5]. An EB model predicts that rates of morphological diversification have decreased exponentially through time [7]. The SURFACE algorithm produces models of varying complexity that assume that evolution has been constrained towards different adaptive peaks along different branches in the phylogeny [4]. BM, OU, and EB models were fit using the function “fitContinuous” in the R package “geiger” [67].

DTT computes the morphological disparity of each subclade relative to the morphological disparity of all taxa, and plots the change in average relative subclade disparity through time. The observed pattern can be compared to the expected pattern under any evolutionary model by simulating data under the desired evolutionary model and then performing DTT analyses on the simulated datasets. The area between the curves representing the observed and simulated changes in disparity, also known as the morphological disparity index (MDI), is used to assess the similarity of observed and expected patterns of phenotypic divergence. Relative subclade disparity is expected to decrease linearly towards the present if phenotypic evolution has occurred under a constant rate process. In contrast, a rapid drop in subclade disparity near the base of the tree is interpreted as early accumulation of phenotypic disparity [38].

We performed DTT analyses on the 1000 posterior distribution trees and compared each to 1000 simulations of PC1 and PC2 evolution under different evolutionary models using the function “dtt” in the R package “geiger” [67]. Maximum likelihood parameter estimates were used to simulate PC1 scores under a BM, EB, and the SURFACE model and PC2 scores under a single-peak OU model. Several models were compared for PC1 because it has been noted that when a clade includes one or more lineages whose evolutionary trajectory differs from the ancestral mode, such as when a lineage escapes to new adaptive peaks as is the case for PC1 here (see Results), DTT is better at detecting early bursts than maximum likelihood approaches [39]. DTT plots and simulations were restricted to the lower two thirds of the tree to avoid biases caused by incomplete taxon sampling towards the present [38]. We used a posterior predictive approach to determine whether the observed MDI differed from the various evolutionary expectations of the different evolutionary models [39]. The frequency of simulations with MDI values more extreme than that observed MDI (more positive for + MDI, more negative for –MDI) was used to compare the ability of different models to predict the observed pattern of phenotypic divergence [38,68].

### Ethics

This study did not require and Animal Use Protocol. We used preserved museum specimens deposited in ROM, Museu de Ciências e Tecnologia da PUCRS, and ANSP collections. No specimens were collected specifically for this study.

## Availability of supporting data

The MCC tree, 1000 chronogram set, and body size and morphometric data supporting the results of this article are available in the Dryad repository, http://datadryad.org/review?doi=doi:10.5061/dryad.vm263 [61].

## Additional files

Additional file 1:
**Locomotor Attributes Details.** Measurement details and functional and ecological implications of locomotor attributes used in this study. Additional file 2:
**Specimen Details.** Number of specimens and range of body sizes of species for which morphometric data was collected. Additional file 3:
**Diagram of** ***Geophagus*** **specimen.** A diagrammatical representation of a cichlid in left-lateral and frontal views showing how locomotor attributes were measured.

## Abbreviations

BM: Brownian Motion
CI: Confidence interval
DTT: Disparity through time
EB: Early burst
MCC: Maximum Clade Credibility
OU: Ornstein-Uhlenbeck
PC: Principal Component
PCA: Principal Components Analysis
ROM: Royal Ontario Museum
SD: Standard deviation

## References

[1] Simpson G (1953). The major features of evolution.

[2] Schluter D (2000). The Ecology of Adaptive Radiation.

[3] Gavrilets S, Losos JB (2009). Adaptive radiation: contrasting theory with data. Science.

[4] Butler MA, King AA (2004). Phylogenetic comparative analysis: a modeling approach for adaptive evolution. Am Nat.

[5] Hansen TF (1997). Stabilizing selection and the comparative analysis of adaptation. Evolution.

[6] Hansen TF, Svensson E, Calsbee R (2012). Adaptive landscapes and macroevolutionary dynamics. The adaptive landscape in evolutionary biology.

[7] Harmon LJ, Losos JB, Davies TJ, Gillespie RG, Gittleman JL, Jennings BW (2010). Early bursts of body size and shape evolution are rare in comparative data. Evolution.

[8] Dornburg AB, Sidlauskas B, Santini F, Sorenson L, Near RJ, Alfaro M (2011). The influence of an innovative locomotor strategy on the phenotypic diversification of triggerfish (family: Balistidae). Evolution.

[9] López-Fernández H, Arbour JH, Winemiller KO, Honeycutt RL (2013). Testing for ancient adaptive radiations in neotropical cichlid fishes. Evolution.

[10] Colombo M, Damerau M, Hanel R, Salzburger W, Matschiner M. Diversity and disparity through time in the adaptive radiation of Antarctic notothenioid fishes. J Evol Biol. 2014; doi:10.1111/jeb.12570. 10.1111/jeb.12570 PMC4407914 25495187

[11] Eastman JM, Wegmann D, Leuenberger C, Harmon LJ. Simpsonian “evolution by jumps” in an adaptive radiation of *Anolis* lizards. ArXiv preprint. 2013: arXiv:1305.4216 [q-bio.PE].

[12] Mahler LD, Ingram TI, Revell LJ, Losos JB (2013). Exceptional convergence on the macroevolutionary landscape in island lizard radiations. Science.

[13] Arnold SJ (2014). Phenotypic evolution: the ongoing synthesis (American society of Naturalists address). Am Nat.

[14] Arbour JH, López-Fernández H (2014). Adaptive landscape and functional diversity of Neotropical cichlids: implications for the ecology and evolution of Cichlinae (Cichlidae; Cichliformes). J Evol Biol.

[15] Losos JB (2010). Adaptive radiation, ecological opportunity, and evolutionary determinism. Am Nat.

[16] Glor RE (2012). Phylogenetic insight on adaptive radiation. Annu Rev Ecol Evo Syst.

[17] Grant PR, Grant RB (2002). Adaptive radiation of Darwin’s finches. Am Sci.

[18] Monteiro LR, Nogueira MR (2011). Evolutionary patterns and processes in the radiation of phyllostomid bats. BMC Evol Biol.

[19] Cooper WJ, Parsons K, Mclntyre A, Kern B, McGee**-**Moore A, Albertson RC. Bentho-pelagic divergence of cichlid feeding architecture was prodigious and consistent during multiple adaptive radiations within African rift-lakes. PLoS ONE*.* 2010; doi:10.1371/journal.pone.0009551. 10.1371/journal.pone.0009551 PMC2833203 20221400

[20] Arbour JH, López**-**Fernández H. Ecological variation in South American geophagine cichlids arose during an early burst of adaptive morphological and functional evolution. Proc R Soc B. 2013; doi:10.1098/rspb.2013.0849 10.1098/rspb.2013.0849 PMC3774233 23740780

[21] Irschick DJ. Evolutionary approaches for studying functional morphology. examples from studies of performance capacity. 2002;42:278–8 10.1093/icb/42.2.278 21708719

[22] Webb PW (1984). Body form, locomotion and foraging in aquatic vertebrates. Amer Zool.

[23] Wainwright PC, Bellwood DR, Westneat MW (2002). Ecomorphology of locomotion in labrid fishes. Environ Biol Fish.

[24] Higham TE (2007). The integration of locomotion and prey capture in vertebrates: morphology, behavior and performance. Integr Comp Biol.

[25] Webb PW (1984). Form and function in fish swimming. Sci Am.

[26] Walker JA, Westneat MW (2002). Performance limits of labriform propulsion and correlates with fin shape and motion. J Exp Biol..

[27] Langerhans RB, Reznick DN, Domenici P, Kapoor BG (2010). Ecology and evolution of swimming performance in fishes: predicting evolution with biomechanics. Fish locomotion: an eco-ethological perspective.

[28] Price SA, Tavera JJ, Near TJ, Wainwright PC (2012). Elevated rates of morphological and functional diversification in reef-dwelling Haemulid fishes. Evolution.

[29] Winemiller KO, Kelso-Winemiller LC, Brenkert AL (1995). Ecomorphological diversification and convergence in fluvial cichlid fishes. Environ Biol Fishes..

[30] López-Fernández H, Honeycutt RL, Winemiller KO (2005). Molecular phylogeny and evidence for an adaptive radiation of geophagine cichlids from South America (Perciformes: Labroidei). Mol Phylogenet Evol.

[31] López**-**Fernández H, Winemiller KO, Montaña C, Honeycutt RL. Diet-morphology correlations in the radiation of South American geophagine cichlids (Perciformes: Cichlidae: Cichlinae). PLoS ONE. 2012; doi:10.1371/journal.pone.0033997. 10.1371/journal.pone.0033997 PMC3317448 22485154

[32] López-Fernández H, Arbour JH, Willis SC, Watkins C, Honeycutt RL, Winemiller KO. Morphology and efficiency of a specialized foraging behavior, substrate sifting, in Neotropical cichlid fishes. PLoS ONE. 2014; doi:10.1371/journal.pone.0089832. 10.1371/journal.pone.0089832 PMC3945966 24603485

[33] Montaña CG, Winemiller KO (2013). Evolutionary convergence in Neotropical cichlids and Nearctic centrarchids: evidence from morphology, diet, and stable isotope analysis. Biol J Linn Soc.

[34] Montaña CG, Winemiller KO (2010). Local-scale habitat influences morphological diversity of species assemblages of cichlid fishes in a tropical floodplain river. Ecol Freshw Fish.

[35] Kullander SO. *Teleocichla*, a new genus of South American rheophilic cichlid fishes with six new species. Copeia. 1988;1998:196–230.

[36] López-Fernández H, Winemiller KO, Honeycutt RL (2010). Multilocus phylogeny and rapid radiations in Neotropical cichlid fishes (Perciformes: Cichlidae: Cichlinae). Mol Phylogenet Evol.

[37] Ingram T, Mahler DL (2013). SURFACE: detecting convergent evolution from comparative data by fitting Ornstein-Uhlenbeck models with stepwise AIC. Methods Ecol Evol.

[38] Harmon LJ, Schulte JA, Larson A, Losos JB (2003). Tempo and mode of evolutionary radiation in iguanian lizards. Science.

[39] Slater GJ, Pennell M (2013). Robust regression and posterior predictive simulation increases power to detect early burst of trait evolution. Syst Biol.

[40] Weihs D (1989). Design features and mechanics of axial locomotion in fish. American Zoologist.

[41] Drucker EG, Lauder GV (2002). Wake dynamics and locomotor function in fishes: interpreting evolutionary patterns in pectoral fin design. Integr Comp Biol.

[42] Drucker EG, Jansen JS (1991). Functional analysis of specialized prey-processing behaviour: winnowing by surfperches (Teleostei: Embiotocidae). J Morphol.

[43] Stayton CT (2008). Is convergence surprising? An examination of the frequency of convergence in simulated datasets. J Theor Biol.

[44] Piálek L, Říčan O, Casciotta J, Almirón, Zrzavý J. Multilocus phylogeny of *Crenicichla* (Teleostei: Cichlidae), with biogeography of the *C. lacustris* group: Species flocks as a model for sympatric speciation in rivers. Mol Phylogenet Evol. 2012;62:46–61. 10.1016/j.ympev.2011.09.006 21971056

[45] Arrington DA (2002). Evaluation of the relationship between habitat structure, community structure, and community assembly in a Neotropical blackwater river.

[46] Standen EM, Lauder GV. Dorsal and anal fin function in bluegill sunfish (*Lepomis macrochirus*): three-dimensional kinematics during propulsion and manoeuvring. J Exp Biol. 2005;208:2753–63. 10.1242/jeb.01706 16000544

[47] Sfakiotakis M, Lane DM, Davies JBC (1999). Review of Fish Swimming Modes for Aquatic Locomotion. IEEE J. Oceanic Eng.

[48] Walker JA, Westeant MW (2010). Mechanical performance of aquatic rowing and flying. Proc R Soc Lond B.

[49] Fulton CJ, Bellwood DR, Wainwright PC (2005). Wave energy and swimming performance shape coral reef fish assemblages. Proc R Soc Lond B.

[50] Zuanon JAS (1999). História natural da ictiogauna de corredeiras do Rio Xingu, na região de altamira, pará.

[51] Klingenberg CP, Ekau W (1996). A combined morphometric and phylogenetic analysis of an ecomorphological trend: pelagization in Antarctic fishes (Perciformes: Nototheniidae). Biol J Linn Soc.

[52] Ingram T, Mahler DL (2011). Niche diversification follows key innovation in Antarctic fish radiation. Mol. Ecol.

[53] Willacker JJ, von Hippel FA, Wilton PR, Walton KM (2010). Classification of threespine stickleback along the benthic-limnetic axis. Biol J Linn Soc Lon.

[54] Hulsey CD, Roberts RJ, Loh YHE, Rupp MF, Streelman JT (2013). Lake Malawi cichlid evolution along a benthic/limnetic axis. Ecol Evol.

[55] Hollingsworth PR, Simsons AM, Fordyce J, Hulsey CD (2013). Explosive diversification following a benthic to pelagic shift in freshwater fishes. BMC Evol Biol.

[56] Near TJ, Dornburg A, Eytan RI, Keck BP, Smith WL, Kuhn KL (2013). Phylogeny and tempo of diversification in the superradiation of spiny-rayed fishes. PNAS.

[57] Friedman M, Keck BP, Dornburg A, Eytan RI, Martin CH, Hulsey CD, Wainwright PC, Near TJ. Molecular and fossil evidence place the origin of cichlid fishes long after Gondwanan rifting. Proc Biol B. 2013; doi:10.1098/rspb.2013.1733. 10.1098/rspb.2013.1733 PMC3779330 24048155

[58] Betancur-R R, Broughton RE, Wiley EO, Carpenter K, Lopez JA, Li C et al. The tree of life and a new classification of bony fishes. PLoS Curr. 2013; doi:10.1371/currents.tol.53ba26640df0ccaee75bb165c8c26288. 10.1371/currents.tol.53ba26640df0ccaee75bb165c8c26288 PMC3644299 23653398

[59] Malabarba MC, Malabarba LR, Del Papa C (2010). Gymnogeophagus eocenicus, n. sp. (Perciformes: Cichlidae), an Eocene Cichlid from the Lumbrera Formation in Argentina. J Vert Paleont.

[60] Malabarba MC, Malabarba, LR, López-Fernández H. On the Eocene cichlids from the lumbrera formation: additions and implications for the Neotropical ichthyofauna. J Vert Paleont. 2014; doi:10.1080/02724634.2013.830021.

[61] Astudillo-Clavijo V, Arbour JH, López-Fernández H. Data from: Divergent selection drove the early diversification of locomotor morphology in the radiation of Neotropical geophagine cichlids. Dryad Digital Repository. 2015. doi:10.5061/dryad.vm263 10.1186/s12862-015-0348-7 PMC4435830 25928151

[62] Rasband WS. Image J. U. S. National Institutes of Health, Bethesda, Maryland, USA. 1997. http://imagej.nih.gov/ij/.

[63] Gerry SP, Wang J, Ellerby DJ (2011). A new approach to quantifying morphological variation in bluegill Lepomis macrochirus. J Fish Biol.

[64] Revell LJ (2011). Phytools: an R package for phylogenetic comparative biology (and other things). Methods Ecol Evol.

[65] Horn JL (1965). A rationale and test for the number of factors in factor analysis. Psychometrika.

[66] Burnham KP, Anderson DR. Model selection and multimodel inference: a practical information**-**theoretic approach. 2nd ed. Berlin: Springer; 2002.

[67] Harmon LJ, Weir JT, Brock CD, Glor RE, Challenger W (2008). GEIGER: investigating evolutionary radiations. Bioinformatics.

[68] Slater GJ, Price SA, Santini F, Alfaro ME (2010). Diversity versus disparity and the radiation of modern cetaceans. Proc R Soc B.

