# Supplementary material for: Selection towards different adaptive optima drove the early diversification of locomotor phenotypes in the radiation of Neotropical geophagine cichlids
Source: BMC Evol Biol. 2015 May 1;15:77. doi: 10.1186/s12862-015-0348-7 (PMC4435830; doi:10.1186/s12862-015-0348-7)
Supplement: Additional file 1: — Locomotor Attributes Details. Measurement details and functional and ecological implications of locomotor attributes used in this study. [file 12862_2015_348_MOESM1_ESM.pdf]

Measurement details and functional importance of morphometric variables.

Trait	Functional Importance	Selected references
Anal fin area (AA)*	Contributes mainly to vertical stability and balance by countering destabilizing forces generated by the body and dorsal fin or by acting as a keel independently of the dorsal fin. This fin is also actively employed in slow swimming gaits. Anal fin area decreases as speed increases.	Breder 1926, Hove et al. 2001, Korsmeye et al. 2002, Standen and Lauder 2005
Body Width : Depth ratio (BWD)‡	BWD=BW/BD. A measure of how laterally compressed a body is. A compressed body facilitates precise turning around the vertical axis.	Webb 1984 b; Weihs 1989
Caudal fin area (CA)*	Contributes to thrust, especially during axial locomotion. A larger surface area moves more water per tail beat and therefore generates more thrust.	Webb 1982, 1984 a, 1984 b; Langerhans 2009; Langerhans and Reznick 2010
Caudal fin aspect ratio (CAR)‡	$CAR=(CH)^2/CA$. A quantitative description of caudal fin shape. High aspect ratio tails are crescent-shaped, which reduces drag and increase swimming efficiency. This is better suited for sustained and high speed swimming, while low aspect ratio tails are better for fast starts/turns and rapid acceleration.	Webb 1982; Weihs 1989; Sfakiotakis et al. 1999; Lauder et al. 2011
Caudal fin height (CH)*	Used to calculate CAR; excluded from comparative analyses.	
Dorsal fin area (DA)*	Contributes to thrust, vertical stability, and balance. Thrust is generated by producing lateral and posterolateral jets. Vertical stability is achieved by producing balancing torques that reduce the tendency to roll or yaw during locomotion or when the fish is not in motion.	Jayne et al. 1996, Drucker and Lauder 2001 a,b, Standen and Lauder 2005
Fineness ratio (FR)‡	FR=SL/BD. Describes how fusiform a body is. A body with a higher FR is more streamline, which helps to minimize drag and maximize thrust. A FR ranging from 4.0-6.5 is optimal for streamlining.	Blake 1983; Chung 2009; Langerhans and Reznick 2010
Frontal area (FA)*	Relates to drag and streamlining. A smaller frontal area contributes to streamlining and produces less drag compared to a larger frontal area.	Langerhans 2009; Lauder et al. 2011

Maximum body depth (BD)†	A deep body reduces lateral recoil by increasing drag and facilitates tight turns by reducing the turning radius around the vertical axis. This improves manoeuvrability but compromises speed and efficiency which are optimized by a shallower, more streamlined body.	Webb 1982 ,1984a, Webb 1984 b; Langerhans 2009
Maximum body width (BW)†	Used to calculate BWD; excluded from comparative analyses.	
Moment arms: pectoral (PcMA), pelvic (PvMA), dorsal (DMA), and anal (AMA) fins*	Distance from the centre of mass to the base of each of the paired and median fins. The moment arm affects the torque the fish produces during swimming. Fish with longer moment arms can produce greater torque and thus require less force to move water, which makes manoeuvres more efficient and precise.	Gatz 1968, Drucker and Lauder 2002; Standen and Lauder 2005
Pectoral fin area (PA)*	Contributes to thrust, especially during foreword and reverse propulsion, turning, and breaking. A large surface area moves more water and thus has a larger effect on the fish's momentum than a smaller surface area.	Webb 1984a, 1984 b; Weihs 1989; Langerhans and Reznick 2010
Pectoral fin aspect ratio (PAR)‡	$PAR=(PL)^2/PA$. A quantitative description of pectoral fin shape. High aspect ratio fins are narrow towards the tip, while low aspect ratio fins are broad. High aspect ratio fins reduces fin-tip vortices, thereby reducing drag relative to lift, which makes them highly efficient. Low aspect ratio fins generate greater thrust during the power stroke.	Gernstner 1999; Sfakiotakis et al. 1999; Walker and Westneat 2000, 2001, 2002; Wainwright et al. 2002. Fisher and Hogan 2007
Pectoral fin length (PL)†	Used to calculate PAR; excluded from comparative analyses.	
Peduncle depth (PD)†	Used to calculate PBD; excluded from comparative analyses.	
Peduncle:Body depth ratio (PBD)‡	Contributes to thrust by increasing water velocity near the trailing edge. A deep peduncle generates more thrust and is thus better for rapid acceleration and fast starts/turns than a shallow peduncle, which is better suited for sustained swimming.	Webb 1982, 1984 b; Fisher and Hogan 2007, Langerhans 2009;
Standard length (SL)†	Measure of body length. Used to correct variables for body size and to calculate FR; excluded from comparative analyses.	

*traits measured on photographs; † traits measured using digital callipers; ‡ trait ratios calculated using measured traits. See additional file 3 for a diagrammatical representation of measurements.

References for Table S2

Blake, R.W. 1983. Functional design and burst-coast-swimming in fishes. *Can. J. Zool.* 61: 2491-2494.

Breder, C. M. 1926. The locomotion of fishes. Zoologica-New York 4, 159-297.

Chung, M.H. 2009. On burst-and-coast swimming performance in fish-like locomotion. *Bioinsp. Biomim.* doi:10.1088/1748-3182/4/3/036001.

Drucker, E. G. and G.V. Lauder. 2001 a. Locomotor function of the dorsal fin in teleost fishes: experimental analysis of wake forces in sunfish. *J. Exp. Biol*. 204: 2943-2958.

Drucker, E. G. and G.V. Lauder. 2001 b. Wake dynamics and fluid forces of turning maneuvers in sunfish. *J. Exp. Biol*. 204: 431-442.

Drucker, E.G. and G.V. Lauder. 2002. Wake dynamics and locomotor function: interpreting evolutionary patterns in pectoral fin design. *Integr. Comp. Biol.* 42: 997-1008.

Gatz, A. 1979. Ecological morphology of freshwater stream fishes. *Tulane Studies in Zoology and Botany* 21: 91-124.

Gerstner, C.L. 1999. Manoeuvrability of four species coral-reef fish that differ in body and pectoral-fin morphology. *Can. J. Zool* 77: 1102-1110.

Gerry, S.P., J. Wang, and D.J. Ellerby. 2001. A new approach to quantifying morphological variation in bluegill *Lepomis macrochirus. J. Fish. Biol.* 78: 1023: 1034.

Fisher, R. and J.D. Hogan. 2007. Morphological predictors of swimming speed: a case study of pre-settlment juvenile coral reef fishes. *J. Exp. Biol.* 210: 2436-2443.

Hove, J. R., L.M. O'Bryan, M.S. Gordon, P.W. Webb D. Weihs. 2001. Boxfishes (Teleostei: Ostraciidae) as a model system for fishes swimming with many fins: kinematics. *J. Exp. Biol.* 204: 1459-1471.

Jayne, B. C., A.F. Lozada G.V. Lauder. 1996. Function of the dorsal fin in bluegill sunfish: motor patterns during four distinct locomotor behaviours. *J. Morphol*. 228: 307-326.

Korsmeyer, K.E., J.F. Steffensen and J. Herskin. 2002. Energetics of median and paired fin swimming, body and caudal fin swimming, and gait transition in parrotfish (Scarus schlegeli) and triggerfish (Rhinecanthus aculeatus). *J. Exp. Biol.* 205: 1253-1263.

Langerhans, R.B. 2009. Trade-off between steady and unsteady swimming underlies predator-driven divergence in *Gambusia affinis. J. Evol. Biol.* 22: 1057:1075.

Langerhans, R.B. and D.N. Reznick. 2010. Ecology and evolution of swimming performance in fishes: predicting evolution with biomechanics. Pp. 200-248 *in* P. Domenici and B.G. Kapoor, eds. Fish locomotion: an eco-ethological perspective. Science Publishers, Enfield, NH.

Lauder, G.V., J. Lim, R. Shelton, C. Witt, E. Anderson and J.L. Tangorra. 2011. Robotic models for studying undulatory locomotion in fishes. *Marine Technology Society Journal* 45: 41-55.

Standen, E.M. and Lauder, G.V. 2005. Dorsal and anal fin function in bluegill sunfish (*Lepomis macrochirus*): three-dimensional kinematics during propulsion and maneuvering. *Journal of Experimental Biology* 208: 2753–2763.

Sfakiotakis, D., D.M. Lane and J.B.C. Davies. 1999. Review of fish swimming modes for aquatic locomotion. *IEEE J. Oceanic Eng.* 24: 237-252.

Wainwright, P.C., D.R. Bellwood and M.W. Westneat. 2002. Ecomorphology of locomotion in labrid fishes. *Environmental Biology of Fishes* 65: 47-62.

Walker, J.A. and M.W. Westeant. 2000. Mechanical performance of aquatic rowing and flying. *Proc. R. Soc. Lond. B* 267: 1875-1881.

Walker, J.A. and M.W. Westneat. 2001. Performance limits of labriform propulsion and correlates with fin shape and motion. *The Journal of Experimental Biology* 205:

Walker, J.A. and M.W. Westeant. 2002. Performance limits of labriform propulsion and correlates with fin shape and motion. *J. Exp. Biol.* 205: 177-187. 177-187.

Webb, P.W. 1982. Locomotor patterns in the evolution of actinopterygian fishes. *Am. Zool.* 22: 329-342.

Webb, P.W. 1984 a. Body form, locomotion and foraging in aquatic vertebrates. *Amer. Zool.* 24: 107-120.

Webb, P.W. 1984 b. Form and function in fish swimming. *Scientific America* 251: 58-68.

Weihs, D. 1989. Design features and mechanics of axial locomotion in fish. *American Zoologist* 29: 151-160.
