# Supplementary material for: Selection towards different adaptive optima drove the early diversification of locomotor phenotypes in the radiation of Neotropical geophagine cichlids
Source: BMC Evol Biol. 2015 May 1;15:77. doi: 10.1186/s12862-015-0348-7 (PMC4435830; doi:10.1186/s12862-015-0348-7)
Supplement: Additional file 2: — Specimen Details. Number of specimens and range of body sizes of species for which morphometric data was collected. [file 12862_2015_348_MOESM2_ESM.pdf]

Number of individuals and the range and mean +/- standard deviation (SD) of standard length (SL) for species used in the study

Genus	Species	Individuals	Min. SL (mm)	Max. SL (mm)	Mean +/- SD SL
Acarichthys	*heckelii*	5	71.01	83.73	75.60 +/- 3.53
Apistogramma	*agassizii*	1	41.68	41.68	41.68 +/- 0.00
Apistogramma	*hoignei*	3	26.52	29.88	28.65 +/- 1.85
Apistogramma	*iniridae*	3	17.07	19.57	19.41 +/- 2.27
Biotoecus	*dicentrarchus*	5	32.28	33.84	33.36 +/- 0.62
Biotodoma	*wavrini*	5	73.58	97.79	84.30 +/- 9.02
Crenicichla	*geayi*	3	72.33	88.68	79.04 +/- 8.56
Crenicichla	*lenticulata*	2	171.67	182.87	177.17 +/- 7.78
Crenicichla	*lugubris*	2	152	188.67	170.33 +/- 25.93
Crenicichla	*multispinosa*	2	209.67	231.097	220.37 +/- 15.13
Crenicichla	*saxatilis*	4	128.93	147.84	140.90 +/- 8.25
Crenicichla	sp. "Orinoco-wallacii"	6	46.23	53.64	50.26 +/- 2.98
Crenicara	*punctulatum*	3	42.56	55.81	49.41 +/- 6.64
Dicrossus	*filamentosus*	4	20.9	25.16	22.71 +/- 1.89
Geophagus	*abalios*	2	75.53	76.06	77.30 +/- 2.49
Geophagus	*dicrozoster*	2	121.45	174.23	147.84 +/- 37.32
"Geophagus"	*steindachneri*	2	94.44	96.29	95.36 +/- 1.31
"Geophagus"	*brasiliensis*	3	111.89	128.23	118.89 +/- 8.41
Guianacara	dacrya	4	87.36	96.91	92.57 +/- 4.34
Guianacara	owroewefi	6	95.1	107.53	100.82 +/- 4.85
Gymnogeophagus	*balzanii*	4	95.7	107.2	101.84 +/- 5.51
Gymnogeophagus	*rhabdotus*	5	65.59	78.56	72.63 +/- 4.83
Mazarunia	*charadrica*	3	66.6	84.41	73.20 +/- 10.59
Mazarunia	*mazarunii*	5	56.59	78.46	65.01 +/- 8.17
Mazarunia	*pala*	3	57.6	74.8	63.41 +/- 9.86
Mikrogeophagus	*altispinosus*	3	36.07	38.11	37.13 +/- 1.02
Mikrogeophagus	*ramirezi*	4	29.01	30.81	29.87 +/- 0.78
Satanoperca	*daemon*	2	137.37	181	159.19 +/- 30.85
Satanoperca	*leucosticta*	4	110.35	162.6	130.02 +/- 22.77
Satanoperca	*jurupari*	4	127.62	151.31	143.96 +/-11.02
Taeniacara	*candidi*	3	32.38	35.96	34.49 +/-1.87
Teleocichla	sp. "preta"	4	85.74	111.97	97.11 +/-10.92
