# Supplementary material for: Selection towards different adaptive optima drove the early diversification of locomotor phenotypes in the radiation of Neotropical geophagine cichlids
Source: BMC Evol Biol. 2015 May 1;15:77. doi: 10.1186/s12862-015-0348-7 (PMC4435830; doi:10.1186/s12862-015-0348-7)
Supplement: Additional file 3: — Diagram of Geophagus specimen. A diagrammatical representation of a cichlid in left-lateral and frontal views showing how locomotor attributes were measured. [file 12862_2015_348_MOESM3_ESM.pdf]

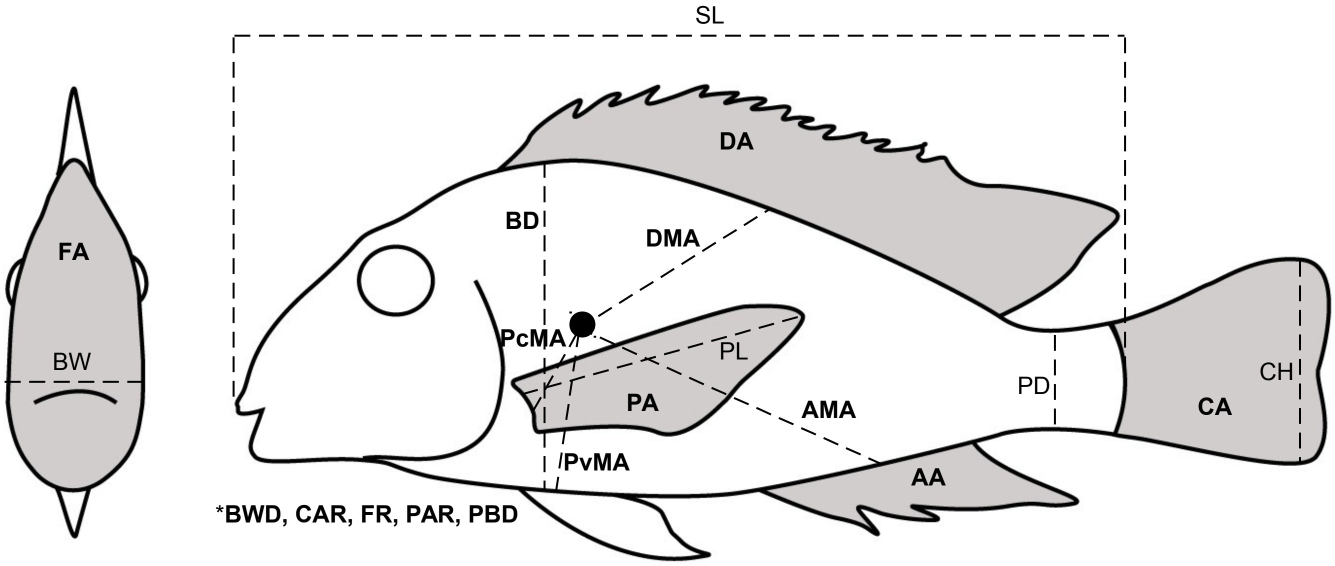

Diagram of *Geophagus* specimen in frontal and left lateral views displaying morphometric measurements. Bolded names correspond to the 15 functional traits used for comparative analyses. Non-bolded names refer to traits that were measured for calculation of trait ratios and were excluded from further analyses. Acronyms correspond to the trait names listed in table S2. * refers to trait ratios not shown on diagram (see additional file 2). Shaded structures indicate surface area measurements. ● represents the centre of mass.
